# Natural Products Repository of Costa Rica (NAPRORE-CR): An Open-Access Database

**DOI:** 10.1021/acs.jcim.5c01552

**Published:** 2025-09-18

**Authors:** Daniel A. Acuña-Jiménez, Jose Rodríguez-Zúñiga, Daniela Gutiérrez-Ramírez, Ricardo Quesada-Grosso, Valery Conejo-López, Kelvin Arce-Villalobos, William J. Zamora, José L. Medina-Franco

**Affiliations:** † DIFACQUIM Research Group, Department of Pharmacy, School of Chemistry, 7180 Universidad Nacional Autónoma de México, Avenida Universidad 3000, Mexico City 04510, México; ‡ CBio3 Laboratory, School of Chemistry, 27915 University of Costa Rica, San Pedro, San José 11501, Costa Rica; § Laboratory of Computational Toxicology and Biological Testing Laboratory (LEBi), 27915 University of Costa Rica, San Pedro, San José 11501, Costa Rica; ∥ Advanced Computing Lab (CNCA), National High Technology Center (CeNAT), Pavas, San José 11501, Costa Rica

## Abstract

Natural products are outstanding sources of bioactive compounds with potential applications not only in drug discovery, but also in the cosmetic industry and natural pesticides. Costa Rica is among the most biologically diverse countries in terms of the number of known species per unit of area, even above conventionally considered megadiverse countries. In this work, we introduce the Natural Products Repository of Costa Rica (NAPRORE-CR), the first dedicated database that compiles structural representations and predicted properties of natural products found and/or characterized in Costa Rica. The first version of this collection comprises 1161 compounds, annotated with structural classifications, calculated structural and physicochemical properties (MW, HBD, HBA, RB, AlogP, and TPSA), and complex descriptors (SA score, QED, nSPS). The diversity and chemical space coverage of compounds in NAPRORE-CR were compared to drugs, pesticides, and cosmetics. Through the analysis and visualization of chemical space coverage and diversity, it was found that NAPRORE-CR has a property profile compatible with applications in all three fields and that its compounds are structurally similar to those of approved drugs and natural pesticides. Cross-referencing NAPRORE-CR with PubChem and ChEMBL, combined with activity predictions, facilitated the identification of both known applications of the included compounds and potential new areas of study. In favor of open science and FAIR principles for data sharing, NAPRORE-CR is freely available at 10.5281/zenodo.7858061.

## Graphical abstract

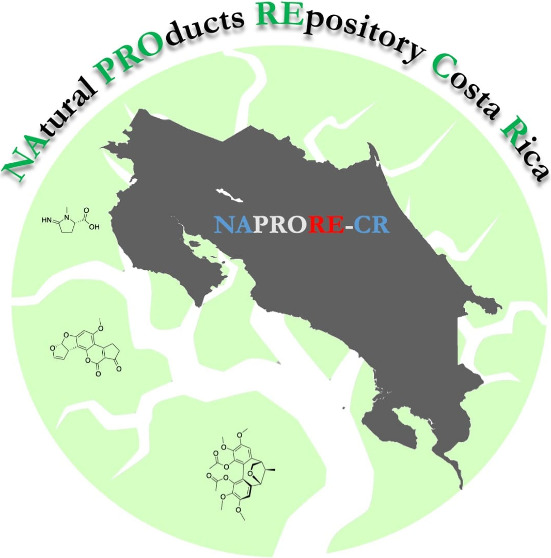

## 1 Introduction

The ever-increasing need to discover novel and effective compounds with potential applications has led to the development of computational methods to aid in the discovery of novel bioactive entities. The primary field benefiting from this technology is drug discovery, where chemoinformatic tools such as virtual screening (VS), quantitative structure–activity relationship models (QSAR), and *in silico* property predictions are utilized to identify lead compounds.^1^ Using these and other techniques encompassed in the field of computer-aided drug design (CADD), more than 70 new drugs have been developed and approved for clinical use.^2^ Although heavily applied in drug discovery, *in silico* methods have also been widely adopted by other industries, such as cosmetics, with QSAR and physiology-based kinetic models for risk assessment of exposure to cosmetic ingredients,^3^ and environmental chemistry, where structure-based molecular design techniques, such as VS and molecular docking, have been applied to find lead compounds for novel pesticide design.^4^

Specialized compound databases are a key component of computer-aided compound research and are a cornerstone in chemoinformatics and artificial intelligence, which depend on high-quality data.^5^ Compound databases provide information on a vast variety of molecules and their properties, from which potential lead compounds with desirable features and activities can be derived.^6^ Public databases such as PubChem,^7^ BindingDB,^8^ ChEMBL,^9^ and ZINC20,^10^ among others, have provided access to valuable data for medicinal chemistry on hundreds of thousands of compounds and their sources.^11^ Other examples in the fields mentioned above include platforms like CCIBP and COSMOS,^12,13^ for information on cosmetic ingredient regulations, physicochemical properties, metabolic routes and *in vitro/in vivo* toxicity; open databases for pesticide discovery, such as the Pesticide Properties Database, Bio-Pesticides Database, and Pesticide-Target Interaction Database;^14^ or even resources relevant to several fields like the Antimicrobial TTC Project, that collects antimicrobial compounds useful as food and cosmetic preservatives, pesticides and others.^15^

Among the compound categorizations found in databases, natural products (NPs) stand out as an abundant source of bioactive molecules of interest. In drug design, during the 1981 to 2019 period, an average of 32% of the new drugs approved each year by the United States Food and Drug Administration (FDA) and other similar governmental organizations worldwide were unaltered NPs, botanical drugs (natural extracts with defined composition,) or compounds derived from NPs, and 28% of the small-molecule therapeutic agents approved at the end of the third quarter of 2019 also fell into these categories.^16^ In cosmetics, market trends indicate a growing interest in products containing NPs with antioxidant, anti-inflammatory, and regenerative properties, among other benefits.^17^ Plants are the primary source of these compounds, with over 5,000 different species being used in cosmetic and cosmeceutical formulations globally.^18^ However, marine organisms have picked up relevance as an alternative source of active ingredients, due to the unique conditions of their ecosystems.^19,20^ In pesticides, NPs represent the source of 21.1% of the new compounds approved for crop protection during the period from 1997 to 2010 by the United States Environmental Protection Agency (EPA), a percentage that increases to 35.7% when including biopesticides (living organisms or substances directly extracted from them with pest control capabilities).^21^ Bacterial compounds have recently surged in interest in this field, representing the source of 42% of first-in-class pesticides in the past 30 years.^22^

Due to the potential applications of NPs, numerous databases have been developed focused on specific regions, industries, source organisms, and compound families. A total of 123 of these resources have been mentioned in literature between 2000 and 2020, of which 92 are open-access and 50 include readily available molecular structures.^23^ Notable mentions include COCONUT, an open NPs repository with 695,133 compounds unified from 63 open-access databases;^24^ SuperNatural 3.0, a freely available database of NPs and derivatives including more than 449,000 entries;^25^ and NP Atlas, a database centered on microbially derived NPs with over 32,000 unique structures.^26^ Many of these databases are centered around region-specific species and knowledge of their use. Examples are the Traditional Chinese Medicine Database at Taiwan, the largest library of NPs obtained in China;^27^ ANPDB, a merged database of natural compounds found in Northern and Eastern Africa;^28^ and IMPPAT, a manually curated database of phytochemicals extracted from Indian medicinal plants.^29^

In Latin America, an enormous, largely unexplored potential lies in the vast natural wealth of the region, estimated to be a third of the global biodiversity.^30^ The biodiversity hotspot status of several areas of the continent (Mesoamerica, Cerrado, Tropical Andes, among others), which means these zones are home to an outstanding number of species while at the same time facing severe loss of habitat, generates urgency to study and conserve these ecosystems as sources for bioactive compounds.^31,32^ In this regard, multiple efforts have been made in Latin American countries to establish repositories of compounds found in their territories. From north to south, examples are BIOFACQUIM (Mexico),^33^ CIFPMA (Panama),^34^ NPDBEjeCol (Colombia),^35^ NuBBE_DB_ (Brazil),^36^ PeruNPDB (Peru),^37^ and NaturAr (Argentina).^38^ It is worth noting the ongoing international effort to create a unified resource encompassing all Latin American NP databases, known as LANaPDB.^39^

Costa Rica is worldwide known for its vast natural wealth, housing close to 100,000 different identified species of plants, fungi, animals, microorganisms, and other taxonomic groups, which represent 4.9% of the total known species on the planet, going up to over a million species and 6% respectively when considering all expected species.^40^ Factoring in its small share of land and marine extension of the globe (0.03% and 0.16% respectively), Costa Rica sits among the most biologically diverse countries in the world in terms of the number of known species per unit of area, even above conventionally considered megadiverse countries such as Brazil and China.^41^ Numerous research projects aimed at the discovery, characterization, identification, and development of potential applications for the species inhabiting its territory and their derived products can be found in the literature. These projects range from novel antibiotic discovery and tumor cell inhibiting compounds, to nanoparticle generation for improved bioavailability and biomass management and utilization. These and several other related lines of work are compiled in Table 1. Chemical structures of representative NPs from Costa Rica are shown in Figure 1. Nonetheless, despite its brimming biodiversity and the evident interest in exploiting its resources, Costa Rica does not currently have a dedicated repository that encompasses the properties and molecular structures of the NPs obtained from species found in the country.

**Table 1:** Examples of Research Lines Related to the Utilization of Natural Products and Resources Found in Costa Rica

**Topic**	**References**
Tumor growth inhibitors	Cruz et al.,^42^ Cao et al.,^43^ Vilariño et al.^44^
Biofilm inhibitors	Park et al.^45^
Novel antibacterial/antibiotic/antiviral agents	Mike et al.,^46^ Tripathi et al.,^47^ Cheng et al.,^48^ Ymele-Leki et al.^49^
Antioxidant/cytotoxic compound extraction	Navarro et al.,^50^ Villalobos-Vega et al.^51^
Natural product biosynthesis	Montero-Zamora et al.^52^
Nanoparticles for property enhancement	Quirós-Fallas et al.,^53^ Castillo-Henríquez et al.,^54^ Araya-Sibaja et al.^55^
Biomass management and utilization	Rosales-López et al.,^56^ Syedd-León et al.^57^
Natural herbicides	Portuguez-García et al.^58^
Food contamination by mycotoxins	Granados-Chinchilla et al.^59^
Characterization of traditional medicinal remedies	Navarro et al.,^60^ Doyle et al.^61^

**Figure 1:**
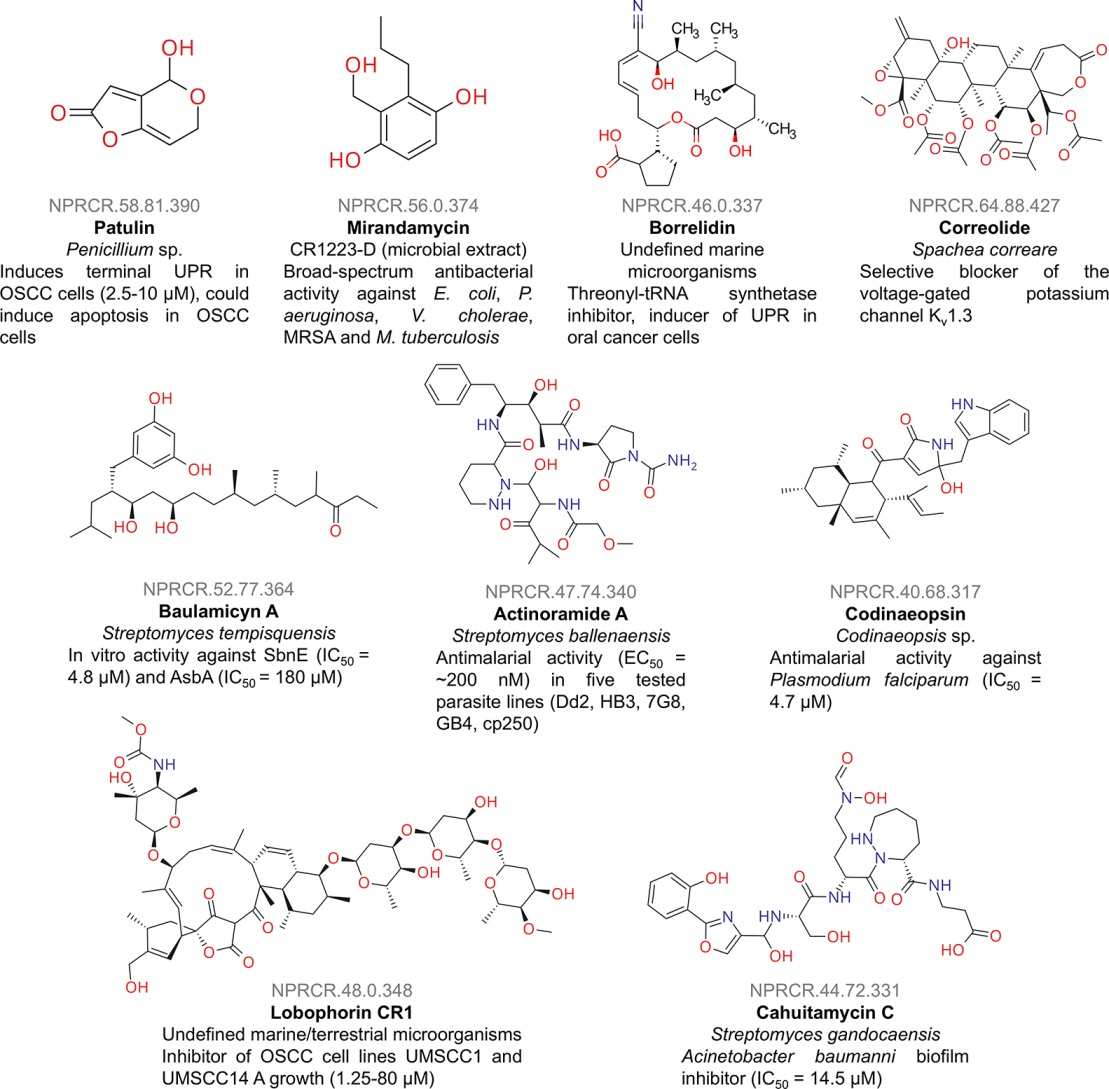
Chemical structures of exemplary natural products from Costa Rica and their reported activities in the scientific literature.

The main objective of this study was to introduce the Natural Products Repository of Costa Rica (NAPRORE-CR) as the first dedicated database that compiles structural representations and predicted properties of NPs found and/or characterized in Costa Rica. The data collection, curation, and standardization processes are described, as well as the compound structural classification, calculation of predicted physicochemical properties, and the characterization and visualization of its chemical space. We compared NAPRORE-CR against representative reference compound sets of approved drugs, cosmetic ingredients, and natural pesticides. The selected reference data sets enable comparisons between the NPs space and the compounds used in these industries, highlighting their prevalence as sources of valuable bioactive compounds.

## 2 Methods

### 2.1 Construction and Curation of the NAPRORE-CR Data Set

NAPRORE-CR was assembled through a literature search of scientific manuscripts related to the discovery, characterization, and application of NPs obtained from native species of the country. This bibliographic research was conducted in three phases: phase one was focused on lines of research developed by researchers of state universities of Costa Rica, mainly from the BIODESS Laboratory at School of Chemistry, Research Centre in Natural Products (CIPRONA), and the Research Centre in Grains and Seeds (CIGRAS) from the University of Costa Rica (UCR), and the Phytochemistry Laboratory (LAFIT) and the Natural Products and Biological Assays Laboratory (LAPRONEB) from the National University of Costa Rica (UNA), selecting only indexed publications that reported NPs obtained from endemic organisms of Costa Rica. The second phase consisted of searches in PubMed, Scopus, and Google Scholar, using the keywords “Costa Rica”, “natural products”, and “University of Costa Rica”, complemented by references cited within those sources. In total, the first two phases included 99 scientific publications.

In the third and last phase, six master’s theses from the Chemistry Postgraduate Programme at the University of Costa Rica were included, covering the extraction, isolation, and structural characterization of NPs. This resulted in 105 documents in the current version of NAPRORE-CR. It is worth mentioning that this is just the first published version of the database, as this repository will be expanded with scientific articles not included or published after the release of this work. One of the future goals of the NAPRORE-CR project is to serve as an updatable and collaborative resource where researchers can deposit new structures as new research is published in the field of NPs from Costa Rica.

The following information was manually mined from the selected manuscripts (when available): compound name, scientific name of the source organism, structural representation, year of publication, and bibliographic information (DOI or permanent link). The structural representations were stored using SMILES strings,^62^ and obtained by searching the PubChem database,^7^ using the compound name as a query for automated requests to its PUG-REST service,^63^ prioritising the isomeric representation when available. Whenever these searches showed no results, the freely available online service DECIMER was employed to generate the corresponding SMILES.^64^ This deep-learning-based tool is capable of transforming an image of a compound’s skeletal formula into several text-based notation formats. All the SMILES generated by DECIMER were manually checked to confirm their fidelity to the sources.

The data set curation was performed using the Python programming language (version 3.10.12), employing the RDKit (version 2024.03.5),^65^ MolVS (version 0.1.1),^66^ and pandas (version 2.2.2) libraries,^67^ for the structure manipulation, standardization, and data management, respectively. The standardization protocol followed widely used protocols previously reported for other NPs databases (e.g., BIOFACQUIM,^33^ NuBBEDB,^36^ PeruNPDB,^37^), as it applied the MolVS default routine to all the SMILES strings, which includes several corrections: normalization of functional groups, recombination of separated charges, breaking of bonds to metal atoms (and elimination of disconnected metallic centers), competitive reionization to ensure the correct ionization order in partially ionized molecules, standardization of stereochemistry information, and validation to identify molecules with unusual and/or problematic characteristics. Several other functions were applied subsequently to remove salts and keep the largest fragment, neutralize charges by addition/removal of hydrogens where possible, ionize partially ionized fragments, and determine a canonical tautomer. It is important to note that this procedure standardizes the tautomeric and ionization states systematically, regardless of prior experimental characterization (e.g., NMR or MS data). In particular, acids are represented in their neutral form by default, following the MolVS protocol. Duplicate entries were removed by generating a numeric identifier for each SMILES, scientific name, and reference, and combining them to create a unique entry ID. The SMILES identifier was also used to combine entries referring to the same compound found in multiple references or species.

### 2.2 Structural Classification (NPClassifier)

The structural classification of the NAPRORE-CR data set was performed using the NPClassifier online service, a freely available deep-learning tool designed to automatically generate a classification for NPs based on an extensive ontology manually constructed from traditional labels found in the literature.^68^ A script was constructed in the Python programming language (version 3.10.12), utilizing the pandas (version 2.2.2) and Requests (version 2.32.3) libraries to make API calls to the NPClassifier server.^69^ The server response includes the assigned categories for every compound at three hierarchical levels: pathway (biosynthetic routes), superclass (metabolite classes, molecular shapes, and/or biosynthetic information), and class (compound families, functional groups, shared scaffolds). The category distribution for every hierarchical level was visually represented in pie charts using the Matplotlib library (version 3.8.0).^70^

### 2.3 Reference Data Sets

#### 2.3.1 Approved Drugs

The FDA-approved drugs data set was obtained from DrugBank, due to the significant presence of NPs in the set and the resource’s relevance as a standard list for drug research. The data set contains 2769 compounds in the version used for this work (5.1.12),^71^ and was last accessed in October 2024 through the DrugBank website (https://go.drugbank.com) with a free Academic License.

#### 2.3.2 Pesticides

A natural pesticides data set was compiled from the “New active ingredient EPA registrations for conventional pesticides from 1997 to 2010” list, as compiled by Cantrell et al.,^21^ and the list of Biopesticide Active Ingredients reported on the official EPA website (https://www.epa.gov),^72^ last accessed in October 2024. The SMILES strings were obtained from PubChem using the compound names as search queries, as described in section 2.1. In the case of mixtures of compounds, such as essential oils, only the main components or those with reported pesticide activity were included. A total of 322 structures were obtained to represent the natural pesticides space.

#### 2.3.3 Cosmetics

The COSMOS TTC data set was selected to represent cosmetic ingredients. This resource comprises cosmetic-related compounds and was created to compile the lowest observed adverse effect levels, based on the concept of the Threshold of Toxicological Concern (TTC).^73^ The total data set includes 966 structures and was last accessed in October 2024 through the official COSMOS website (https://cosmosdb.eu/cosmosdb.v2).

### 2.4 Chemical Property Spaces

#### 2.4.1 Physicochemical and Structural Properties

The property space of the NAPRORE-CR data set was constructed with the following descriptors, commonly used to profile compounds with therapeutic interest:^74^ molecular weight (MW), hydrogen bond donors (HBD) and acceptors (HBA), rotatable bonds (RB), calculated octanol/water partition coefficient (AlogP), and topological polar surface area (TPSA). All these descriptors were generated using the Descriptors module from the RDKit library (version 2024.03.5) for the Costa Rican NPs database and the three reference data sets.

To include a descriptor representative of the ionization profile of the molecules, an acidic (AG) and basic (BG) group counter was constructed using Python (version 3.10.12) with the pandas (version 2.2.2), NumPy (version 1.26.4),^75^ and RDKit libraries. A SMARTS list of acidic and basic functional groups was obtained from AstraZeneca’s Peptide Tools GitHub repository,^76^ which was used to build a script to search for these substructures in all the compounds in the NAPRORE-CR and reference data sets and count the ionizable centers found in each structure. The ionization profile significantly influences properties such as lipophilicity, polarity, and permeability; however, due to the difficulty in accurately determining it, it is often underrepresented in chemical property spaces.^77^ In contrast, it is estimated that up to 77.5% of drugs show ionizable centers, which exacerbates the relevance of the inclusion of this property.^78^

To visualize the value distributions for all the calculated properties, a Python script (version 3.10.12) was constructed using the pandas (version 2.2.2), NumPy (version 1.26.4), Matplotlib (version 3.8.0), and Seaborn (version 0.13.2) libraries. Violin plots were generated for the eight chosen descriptors, with separate graphs for each dataset to visualize their value distributions better.

#### 2.4.2 Synthetic Accessibility, Drug-Like Character, and Molecular Complexity

The Synthetic Accessibility Score (SAScore) was included as a preliminary assessment of the synthetic feasibility of the studied compounds. Compounds with an SAScore ≤ 6 are considered synthetically accessible.^79^ The quantitative estimate of drug-likeness (QED) serves as a measurement of drug-likeness based on the calculated physicochemical and structural descriptors of the data set. A QED > 0.67 represents “attractive”, drug-like structures.^80^ Molecular complexity is represented by the size-normalized spacial score (nSPS), which complements the analysis of the library’s potential as a source of synthetically achievable, drug-like compounds.^81^ An nSPS in the range of 10–20 coincides with most FDA-approved drugs, as recommended by the authors of this scoring system.^82^ All three descriptors were calculated using Python (version 3.10.12) with the RDKit library (version 2024.03.5), alongside pandas (version 2.2.2) for data manipulation. Histograms were generated for each of these descriptors to visualize their value distributions, using Matplotlib (version 3.8.0) and Seaborn (version 0.13.2).^83^

### 2.5 Commercial Availability

Commercial availability of the NAPRORE-CR compounds was determined using the PUG-REST service to retrieve chemical vendor information. A Python (version 3.10.12) script was constructed using the urllib module and the Requests (version 2.32.3) and pandas (version 2.2.2) libraries to cross-reference NAPRORE-CR compounds with PubChem to obtain their CIDs, which were then used as a search term to find all available vendors included in PubChem for each compound. The number of unique vendors was counted and stored when data was available; otherwise, a value of zero was reported when no results were found or no CID could be obtained.

### 2.6 Bioactivity Profile

#### 2.6.1 Experimental Bioactivity Profile

The ChEMBL API was consulted via a Python script (version 3.10.12) to cross-reference with the NAPRORE-CR database and retrieve the ChEMBL ID of the available compounds, utilizing the chembl_webresource_client library (version 0.10.9),^84^ alongside Requests (version 2.32.3). The obtained IDs were then used to retrieve all the available activity data for the Costa Rican NPs using the *activity* module. A promiscuity ratio was calculated by dividing the number of activity assays marked as Active under the “*activity_comment*” section by the total number of activity assays found for every compound. This metric was then used to represent the bioactivity profile of the NAPRORE-CR molecular scaffolds in a constellation plot.^85^

#### 2.6.2 Predicted Bioactivity Profile (Target Fishing)

To explore the potential of studying specific biological targets, the NetInfer web server was utilized for an inverse virtual screening (target fishing) analysis.^200^ NetInfer works via network-based inference methods to make affinity predictions based on bioactivity data from ChEMBL and BindingDB chemical substructures, by associating them with substructures extracted from a training set and comparing them to the input compounds in the SMILES format. The website (https://lmmd.ecust.edu.cn/netinfer) allows for batch processing and generates a report with information related to the top-ranked targets based on a scoring function correlated with binding affinities. In this study, the weighted substructure-drug target network-based inference method was used with the default parameters and configuration (Morgan fingerprints, α = 0.4, β = 0.2, γ = −0.5, δ = 20, ε = 4, k = 2, 50 predictions per compound) to get the top 50 protein targets with the best predicted affinities for every compound. The list was further shortened to the top 5 results per compound, and their gene symbols and protein families were used to construct a frequency bar plot using Python (version 3.10.12) with the pandas (version 2.2.2) and Matplotlib (version 3.10.3) packages. The *target* module from the chembl_webresource_client library (version 0.10.9) was used to retrieve the UniProt identifier for the targets found in the NAPRORE-CR experimental activity results and match them with the NetInfer results to identify coincidences between predicted and experimental activity profiles.

### 2.7 Chemical Space Visualization

To generate visualizations of the chemical space of NAPRORE-CR and the reference data sets, principal component analysis (PCA) was chosen to represent the physicochemical and structural descriptor space, and Tree MAPs (TMAPs) for the structural similarity space. PCA uses the original high-dimensional space variables to generate new, uncorrelated components by linearly combining them, thereby maximizing the data’s variability explained by each component.^86^ The TMAPs algorithm generates minimum spanning trees to better represent the structural relationships between compounds in large data sets, preserving both global and local features for interpretation.^87^

#### 2.7.1 PCA

The same libraries and versions mentioned in Section 2.4.1 were used, along with the SciKit-learn library (version 1.5.2), for PCA.^88^ A cumulative explained variance graph was generated to choose the number of principal components necessary to represent at least 70% of the data sets’ variance; this threshold was chosen because it is a common cutoff value in this type of analysis.^89^ Finally, the PCA graphs were generated for NAPRORE-CR and all sets combined, with their respective descriptor vectors plotted on top of a scatter plot. The dots in each graph are colored to represent the respective pathway (NAPRORE-CR) or data set (combined sets) of the corresponding compound.

#### 2.7.2 TMAP

A Python (version 3.9.20) virtual environment was set up to generate the tree MAPs, utilizing the TMAP (version 1.0.6), pandas (version 2.2.3), Numpy (version 1.26.4), Matplotlib (version 3.9.2), Seaborn (version 0.13.2), and RDKit (version 2024.3.5) libraries. MACCS keys for all the compounds were generated with RDKit’s MACCSkeys module. A 166-bit MinHash function was applied to the fingerprint vectors, and an LSHForest with 128 iterations was generated from them. A k-nearest neighbors search was conducted with k = 10 for the NAPRORE-CR set and k = 20 for the combined sets. These values were chosen following the examples found in the TMAP library documentation and considering the number of compounds in each space. A list of weighted vectors obtained from the neighbors search was generated to create a graph, which was then used to find the minimum spanning tree and set the layout for the final TMAP. Colors were assigned to each data point to represent pathways (NAPRORE-CR) or data sets (combined sets).

#### 2.7.3 Constellation Plot

The Constellation Plot was created as previously proposed, utilizing the “*get-cores.py*” program to wash the NAPRORE-CR structures, extract the cores of every molecule, and generate the analog series, employing custom scripts provided by the authors in the original publication.^85^ After data processing, the “*constellation-plots.ipynb*” notebook was used to generate the t-SNE low-dimensional chemical space and produce the final plot, using the following modules and libraries: Math, Itertools, Scripts, RDKit (version 2024.03.5), NumPy (version 2.2.6), pandas (version 2.2.3), SciKit-learn (version 1.5.2), and Matplotlib (version 3.10.3). The following default parameters were used in the t-SNE function: perplexity = 40, iterations = 3000, minimum molecules per core = 2. The promiscuity ratio generated with the activity assay information extracted from ChEMBL (section 2.4) was used to map the average promiscuity of every core cluster onto the constellation plot.

### 2.8 Global Diversity Analysis

To assess and compare the diversity of the compound data sets using multiple representations, a Consensus Diversity Plot (CDP) was generated as described by González-Medina et al.^90^ A Python script (version 3.10.12) was built with the same libraries and versions mentioned in section 2.4.2, plus RDKit (version 2024.03.5) and SciPy (1.13.1).^91^ Tanimoto coefficients were calculated for each possible pair of compounds in each data set using their MACCS keys, and a mean value was calculated for each data set. Murcko scaffolds were generated using RDKit and used to group the compounds in each data set by their common scaffolds. The groups were ranked in descending order by number of compounds, and the cumulative fraction of scaffolds was calculated. Cyclic system recovery curves were built for each data set, and their corresponding area under the curve (AUC) was obtained as a measure of scaffold diversity. The physicochemical and structural descriptors generated in section 2.2 were normalized to calculate the mean intraset Euclidean distance of the property space for each data set. The CDP was constructed by plotting the mean Tanimoto coefficients on the *X*-axis, the AUC values on the *Y*-axis, and the mean intraset Euclidean distances of the descriptors as a continuous color scale. Each data set was represented as a dot, the size of which was proportional to the number of included compounds.

## 3 Results and Discussion

### 3.1 Database Construction and Curation

From the 105 manuscripts included in the current version of NAPRORE-CR, which span the 1988–2024 period, a total of 2121 compound entries were extracted prior to data curation and merging of duplicate entries. After applying the curation protocol described in Section 2.1, the compound list was reduced to 1161 entries, including stereoisomers identified in the consulted literature. The number of compounds retained and discarded in each data set after applying the molecular weight filter is shown in Table 2, corresponding to the sets used for the subsequent chemical space analysis. The complete NAPRORE-CR data set can be freely accessed through its Zenodo repository at 10.5281/zenodo.7858061.^92^

**Table 2:** Number of Compounds Per Data Set after Filtering

**Data set**	**Total compounds**	**M** **W < 1500** **Da**	**Filtered out**
NAPRORE-CR	1161	1161	0
DrugBank	2769	2521	248
EPA	322	320	2
COSMOS	966	961	5

### 3.2 Structural Classification (NPClassifier)

The compounds included in NAPRORE-CR show representation of the seven biosynthetic pathways of the NPClassifier ontology, as well as 44 of its superclasses and 170 of its classes; distributions by level are shown in Figures 2 and 3. The most frequently found pathways, arranged in decreasing order of frequency, were terpenoids (48.7%), followed by shikimate-phenylpropanoids (22.7%) and fatty acids (12.0%). The most represented superclasses were sesquiterpenoids (28.9%), monoterpenoids (10.6%), and flavonoids (9.0%). Lastly, the most common classes were germacrane sesquiterpenoids (7.3%), cadinane sesquiterpenoids (4.3%), and wax monoesters (4.3%). These observations are consistent with the sources used to build the database, as these were primarily studies on the phytochemistry and biochemistry of marine microorganisms. The high presence of terpenoids in the database was expected, as the cyclization reactions of polyolefinic carbocations found in plant metabolism give rise to over 20000 polycyclic base structures.^93^ This great structural diversity has been valuable in the discovery of antiparasitic compounds throughout history, such as the case of artemisinin and malaria.^94^ Shikimate-phenylpropanoids are another common compound type in NP databases with a high presence of plant-derived compounds, like NAPRORE-CR, as they are vital for pest resistance (tannins), reproduction (pigments such as flavonoids and anthocyanins), and structural stability (biopolymers such as lignin and suberin) of plants.^95^ Finally, the notable presence of fatty acids in the database can be associated with the multiple studies on essential oils of several plant genera (e.g., *Croton, Lippia, Ocotea*) and compound extraction from fruits such as berries (e.g., *Piper nigrum, Psidium friedrichsthalianum).* A small portion of compounds could not be classified by NPClassifier (2.2%), with most structures corresponding to benzofurans and phenolic compounds.

**Figure 2:**
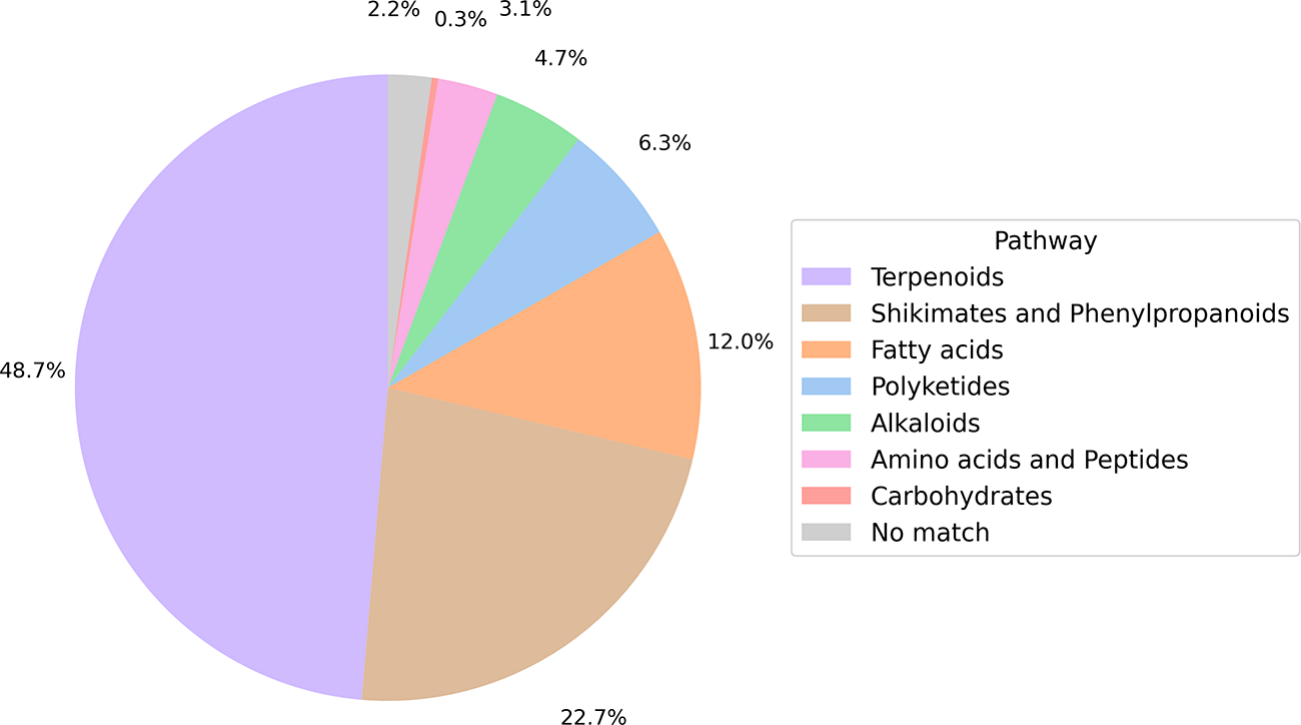
Structural classification of NAPRORE-CR compounds by biosynthetic pathway, according to the NPClassifier ontology.

**Figure 3:**
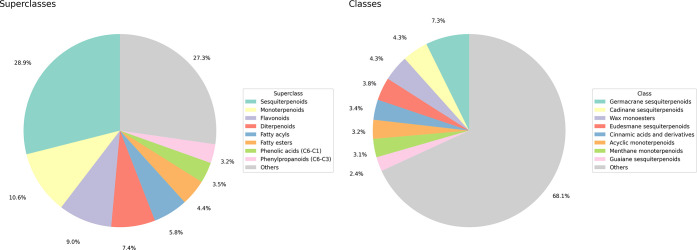
Structural classification of NAPRORE-CR compounds according to the NPClassifier ontology: a) Superclasses; b) Classes.

The predominant superclasses follow the same pattern observed in the biosynthetic pathways, since they correspond to subcategories of terpenoids (sesquiterpenoids, monoterpenoids, diterpenoids), shikimate-phenylpropanoids (flavonoids, phenolic acids, phenylpropanoids), and fatty acids (fatty acyls, fatty esters). The most frequently found classes (sesquiterpenes and wax monoesters) denote the presence of a high number of compounds derived from essential oils and volatile fractions of Costa Rican flora studied by Cicció,^96–98^ which comprise a notable portion of the manuscripts included in the current version of NAPRORE-CR. Previous analysis of the LANaPDB database, which included a preliminary set of NAPRORE-CR compounds, revealed the predominance of certain fragment combinations in the molecules of the Costa Rican NP space, suggesting an uneven representation of plants versus other types of organisms (e.g., bacteria, fungi, animals). Future updates should aim to diversify the chemical space by incorporating compounds derived from species found in other taxonomic kingdoms, thereby improving its representativeness.

### 3.3 Chemical Property Spaces

#### 3.3.1 Physicochemical and Structural Properties

Figure 4 shows the value distributions of the selected properties to represent the size (MW), flexibility (RB), polarity (AlogP, TPSA, HBA, HBD), and ionizability (AG, BG) of the compounds in the NAPRORE-CR and reference spaces. The MW plots showed that the NAPRORE-CR compounds have a value distribution more similar to the pesticides and cosmetics reference sets, in contrast with the approved drugs set. The median and mean values of NAPRORE-CR (250.2 g/mol and 303.1 g/mol, respectively) are the second highest of the analyzed sets, second only to DrugBank (325.5 g/mol and 361.0 g/mol, respectively). The abundance of molecules with a higher MW in the approved drugs data set might be derived from the presence of synthetic compounds with complex and diverse structures. This reflects a predominance of relatively simple scaffolds, such as terpenoids and phenylpropanoids isolated from essential oils and plant extracts, in the Costa Rican NP space, which are typically smaller and less structurally modified than synthetic drugs. These structural features explain the closer similarity of NAPRORE-CR compounds to cosmetic ingredients and natural pesticides, where such scaffolds are also common. Overall, NAPRORE-CR compounds are more similar to the cosmetic and natural pesticide spaces in terms of molecular size.

**Figure 4:**
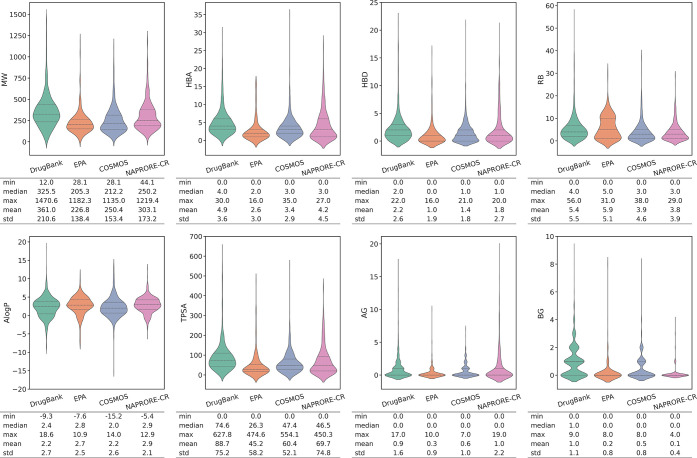
Violin plots for the selected structural and physicochemical descriptors of NAPRORE-CR and the reference data sets (*n* = 2521 DrugBank; 320 EPA; 961 COSMOS, 1161 NAPRORE-CR). Dotted lines indicate the first and third quartiles, dashed lines indicate the median values. A summary of statistical parameters is included below each plot. MW: molecular weight; HBA: hydrogen bond acceptors; HBD: hydrogen bond donors; RB: rotatable bonds; AlogP: atomic octanol/water partition coefficient (calculated); TPSA: total polar surface area; AG: acidic groups; BG: basic groups.

In terms of flexibility, the RB violin plots showed that NAPRORE-CR has a similar distribution and central tendency measures (median 3.0; mean 3.8) to the COSMOS database compounds (median 3.0; mean 3.9). Both of these data sets showed their maximum frequency near the first quartile and a unimodal distribution. Although it was expected to find a similar distribution in the EPA data set, as it is composed of NPs used as pesticides, it showed a bimodal distribution with a more uniform value distribution up to 15 RB. This result could be traced to the low sample size of this data set, as it was the smallest space analyzed, which might have resulted in an underrepresented chemical space.

Referring to the polarity measurements, the AlogP showed a higher similarity between the NAPRORE-CR space and the natural pesticides data set, as their central tendency measures are the closest among the compared sets (NAPRORE-CR: median 2.9; mean 2.9; EPA: median 2.8; mean 2.7). As previously commented, this was expected, as these two sets are entirely composed of NPs. The TPSA descriptor showed similar value profiles for NAPRORE-CR and COSMOS, characterized by a low mode, a high frequency band exceeding 100 Å^2^, and close median values (NAPRORE-CR: 46.5 Å^2^; COSMOS: 47.4 Å^2^). For the third and last polarity measure, the HBA and HBD plots for NAPRORE-CR were most similar to the DrugBank ones, with higher frequencies in high HBA values (>10 acceptors) that gave them higher mean values (DrugBank: 4.9; EPA: 2.6; COSMOS: 3.4; NAPRORE-CR: 4.2) compared to the other sets, and similar distributions and mean values for HBD as well (DrugBank: 2.2; NAPRORE-CR: 1.8). Depending on the selected individual descriptor, all reference data sets have similar polarity profiles when compared to NAPRORE-CR.

Ionizability showed certain similarities between NAPRORE-CR and DrugBank in terms of their acidic group counts, while the EPA set is closer in terms of basic group counts. While the similarity between NAPRORE-CR and EPA can be attributed once again to the origin of their compounds, the differences with DrugBank can be explained by the structural modifications commonly performed on drug candidates to improve properties such as ligand affinity. By adding basic functional groups, these can interact with acidic amino acid residues in the protein’s active site.^99^ Furthermore, the biosynthetic routes found in the NAPRORE-CR compounds related to basic moieties were mainly alkaloids, polyketides, and amino acids and peptides, which together represent only a minor fraction of the data set. Notably, the standard deviation values for these properties were significantly larger than their corresponding mean values, making it inadequate to draw conclusive observations; however, it is worth noting that the low abundance of ionizable groups in NAPRORE-CR directly correlates with classical phytochemical extraction techniques, where the use of nonpolar organic solvents preferentially isolates neutral compounds.

In summary, when comparing the selected descriptors individually, NAPRORE-CR shows similarity with COSMOS’ cosmetic ingredients in terms of size, flexibility, and polarity, and with EPA’s natural pesticides in the molecular weight and AlogP properties. The most dissimilar data set was the approved drugs set, especially in its MW and TPSA distributions. The presence of an essential portion of synthetic and modified molecules could explain the peculiarities of this data set. Nonetheless, the central tendency values for all properties fell under the parameters defined by Lipinski’s rule of five (MW < 500 Da, HBD < 5, HBA < 10, AlogP < 5), which suggests good potential for finding compounds with sufficient bioavailability. The natural pesticides data set’s size resulted in a limitation for the analysis of its properties.

#### 3.3.2 Synthetic Accessibility, Drug-Like Character, and Molecular Complexity

Figure 5 shows the value distributions for the three chosen descriptors to evaluate the synthetic feasibility and drug-likeness of the NAPRORE-CR chemical space. Most of the compounds in the database have SA score values equal to or under 6 (94.92%), indicating that almost the entire set of compounds should be synthetically feasible. The QED score suggests that only a small fraction of compounds (14.21%) exhibit the typical characteristics of a drug-like molecule. This is expected of a set of NPs, as their structural complexity often translates to violations of the common parameters found in already approved drugs, i.e., rule of five. Low QED values do not necessarily mean that this chemical space lacks potential for the exploration of new bioactive compounds, as many NPs are used as drugs despite having one or more violations of these empirical rules; rather, they highlight the importance of further optimization to improve their drug-like character.^100^ The nSPS distribution further emphasizes this observation, as a higher portion of the analyzed compounds (30.15%) showed values in the 10–20 range, associated with most approved drugs, compared to the QED values. When expanding the range to 20–40, as recommended by the authors of this method for exploring promising bioactive compounds with appropriate potency and selectivity, the percentage of included compounds increases to 37.73%, representing a significant proportion of the space.^81^ Together, these results indicate that although Costa Rican NPs often deviate from conventional drug-likeness metrics, they remain synthetically tractable and occupy a region of chemical space comparable to that of known bioactive molecules. This reinforces the relevance of NAPRORE-CR as a source of structurally diverse scaffolds that could inspire lead discovery, particularly when combined with optimization strategies commonly used in medicinal chemistry. By this analysis, it can be concluded that the NAPRORE-CR space shows promising characteristics for the potential synthesis and study of its compounds for medicinal chemistry applications.

**Figure 5:**
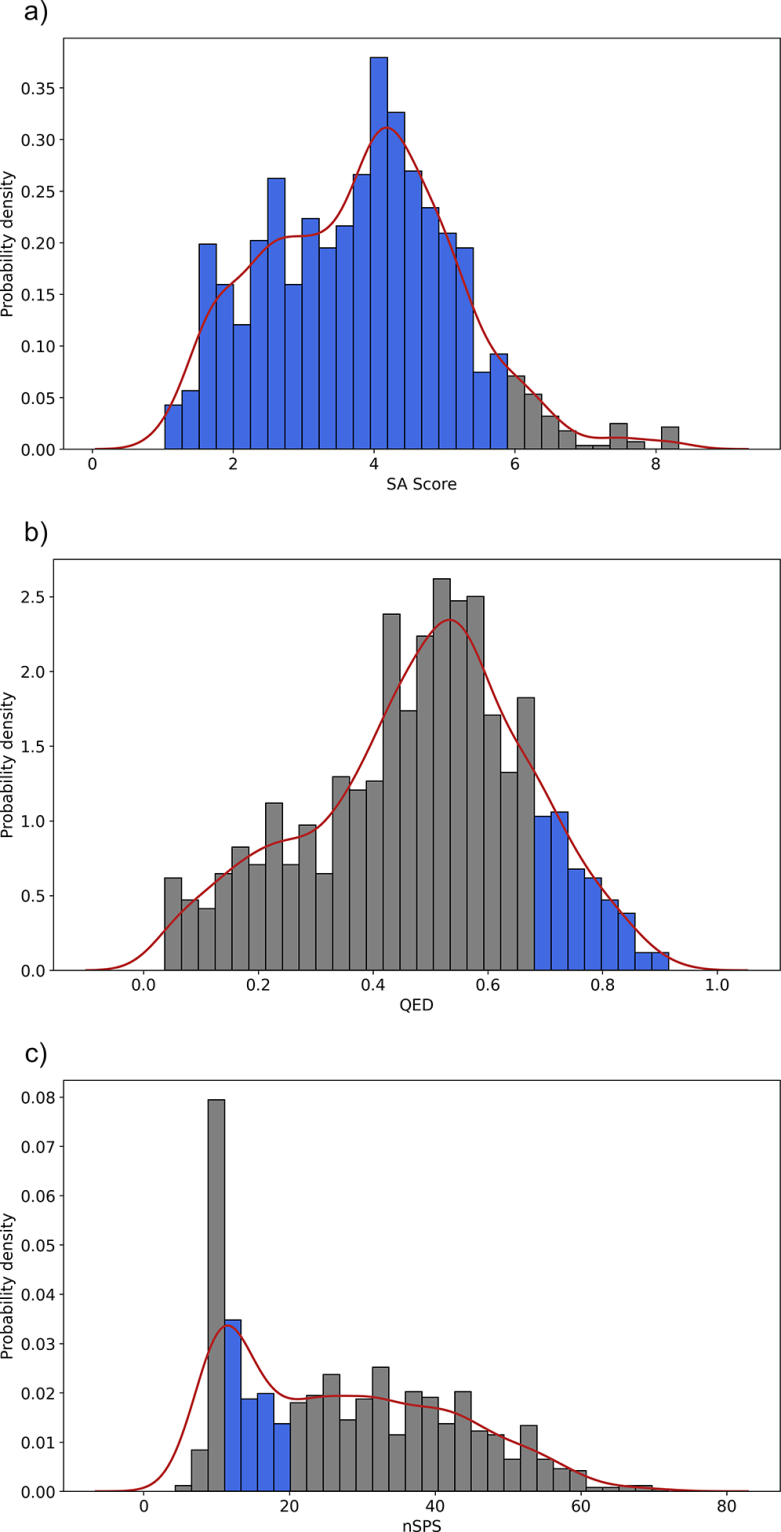
Normalized value distributions for the selected complex descriptors calculated for NAPRORE-CR (*n* = 1161 compounds): a) SA Score; b) QED; c) nSPS. All plots include a histogram (bins = 30) and a kernel density estimate (red line); the highlighted bins correspond to the value ranges associated with favorable synthetic or drug-like characteristics.

### 3.4 Commercial Availability

A total of 751 out of the 1161 compounds in the NAPRORE-CR database had an associated PubChem CID, which is 64.68% of the database. Out of these, 599 had at least one reported chemical vendor, which represents 79.76% of the cross-referenced compounds and 51.59% of the entire data set. The number of unique vendors for every compound can be consulted in the NAPRORE-CR Zenodo repository.^92^

### 3.5 Bioactivity Profile

#### 3.5.1 Experimental Bioactivity Profile

A total of 612 out of 1161 (52.71%) compounds of NAPRORE-CR were cross-referenced with ChEMBL and their IDs retrieved to find activity data. 577 of these molecules had associated activity information (91.01% of ChEMBL-available compounds, 49.70% of NAPRORE-CR), while only 284 had at least one assay reported as active (46.40% of ChEMBL-available compounds, 24.46% of NAPRORE-CR). The entire list of assays retrieved from ChEMBL for NAPRORE-CR compounds can be freely accessed from its Zenodo repository.^92^

#### 3.5.2 Predicted Bioactivity Profile (NetInfer-Based Target Fishing)

The top 15 protein targets that appeared more frequently in the top five results for every compound were tallied and are presented in Figure 6. The top four targets all correspond to the nuclear hormone receptor family, which suggests great potential for further evaluating the utility of NAPRORE-CR compounds in the study of inhibitors for treating related cancers, such as prostate and breast cancer.^101^ An example of this potential is the top one result, the androgen receptor (AR), which has reports of the usage of NPs present in NAPRORE-CR, such as curcumin, apigenin, quercetin, fisetin, luteolin, kaempferol, berberine, eugenol, gingerol and ellagic acid, for treatment of castration resistance in patients with advanced stage prostate cancer.^102^ As this is just a proof-of-concept to explore the possible applications of the database, the complete results sheet for the target fishing is available on the NAPRORE-CR Zenodo repository for further scrutiny.

**Figure 6:**
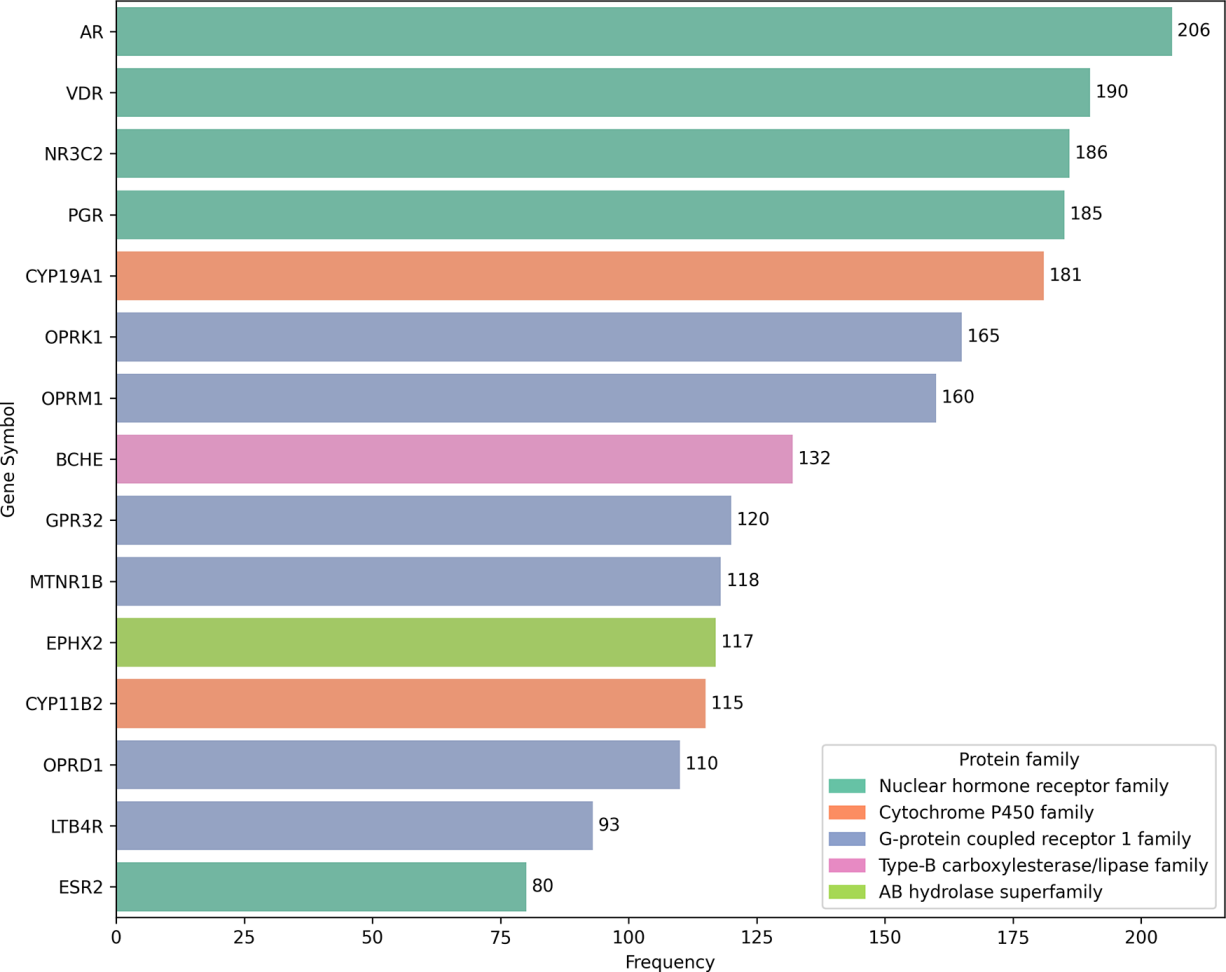
Frequency of occurrence of protein targets in binding affinity predictions of NAPRORE-CR compounds by the NetInfer web service. Colors correspond to protein family.

As shown in Table 3, only three NAPRORE-CR compounds with reported activity in ChEMBL yielded matches with their respective top five predicted matches from the NetInfer service. Fisetin has been reported as an inhibitor of the Aurora B kinase, capable of overriding mitotic arrest and perturbing spindle checkpoint signaling, which causes mitotic inhibition and premature initiation of chromosome segregation in human cancer cells.^103^ Glutamic acid is a nonessential amino acid that acts as an excitatory neurotransmitter, interacting with ligand-gated ion channels (glutamate receptors) in its anionic form (glutamate). When binding to ionotropic receptors, such as the kainate 2 subunit, membrane depolarization is induced in postsynaptic cells, causing signal transmission.^104,105^ Nothofagin has been studied as a selective inhibitor of SGLT2 over SGLT1, to inhibit glucose reabsorption in the kidneys for the treatment of type 2 diabetes, while minimizing adverse effects such as hypoglycemic episodes and urinary tract infections.^106^ Although this initial exploration yielded few matches between experimental and predicted activities, future studies can explore other resources for assay information and target prediction to identify more known applications of Costa Rica’s NPs and potential subspaces that may lead to compounds of medicinal interest.

**Table 3:**
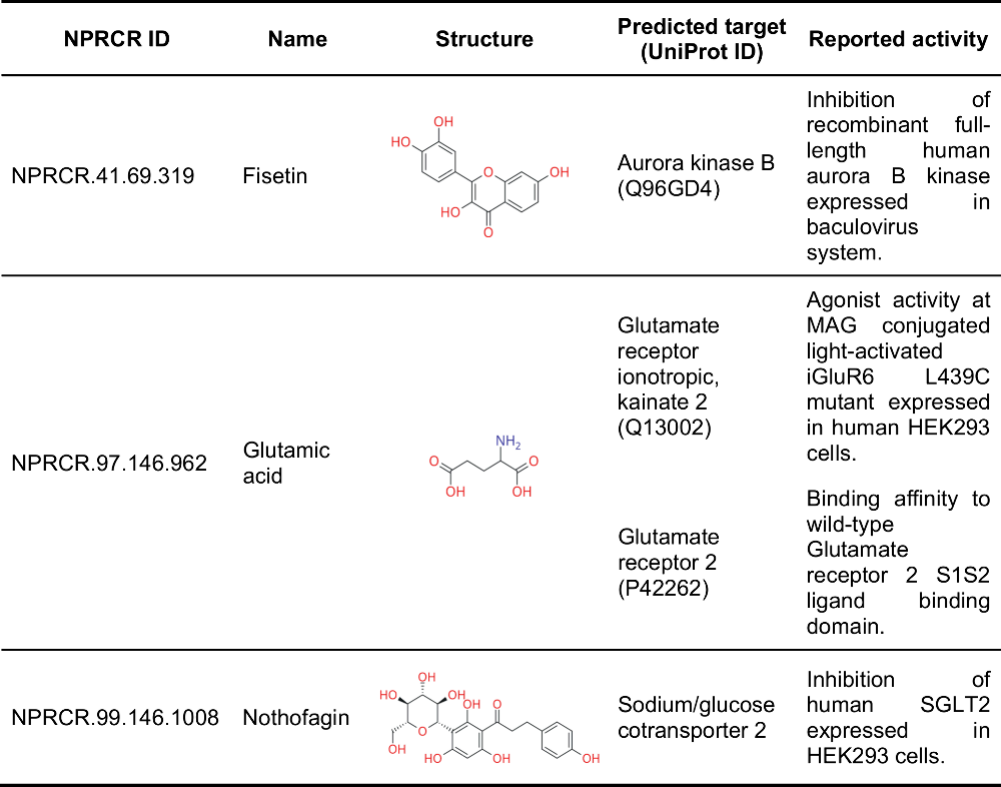
NetInfer Predicted Targets Matched with ChEMBL Reported Activity for NAPRORE-CR Compounds

### 3.6 Chemical Space Visualization

#### 3.6.1 Physicochemical and Structural Properties Similarly (PCA)

The cumulative explained variance plot used to choose the number of dimensions for the PCA of NAPRORE-CR and the combined reference data sets is shown in Figure 7. n = 2 was selected as the optimal number of principal components in both cases, with a cumulative explained variance of 72.47% for NAPRORE-CR and 73.27% for the combined sets. Although adding a third component increased these values to over 80% in both cases, a bidimensional representation was preferred to facilitate visualization and interpretation.

**Figure 7:**
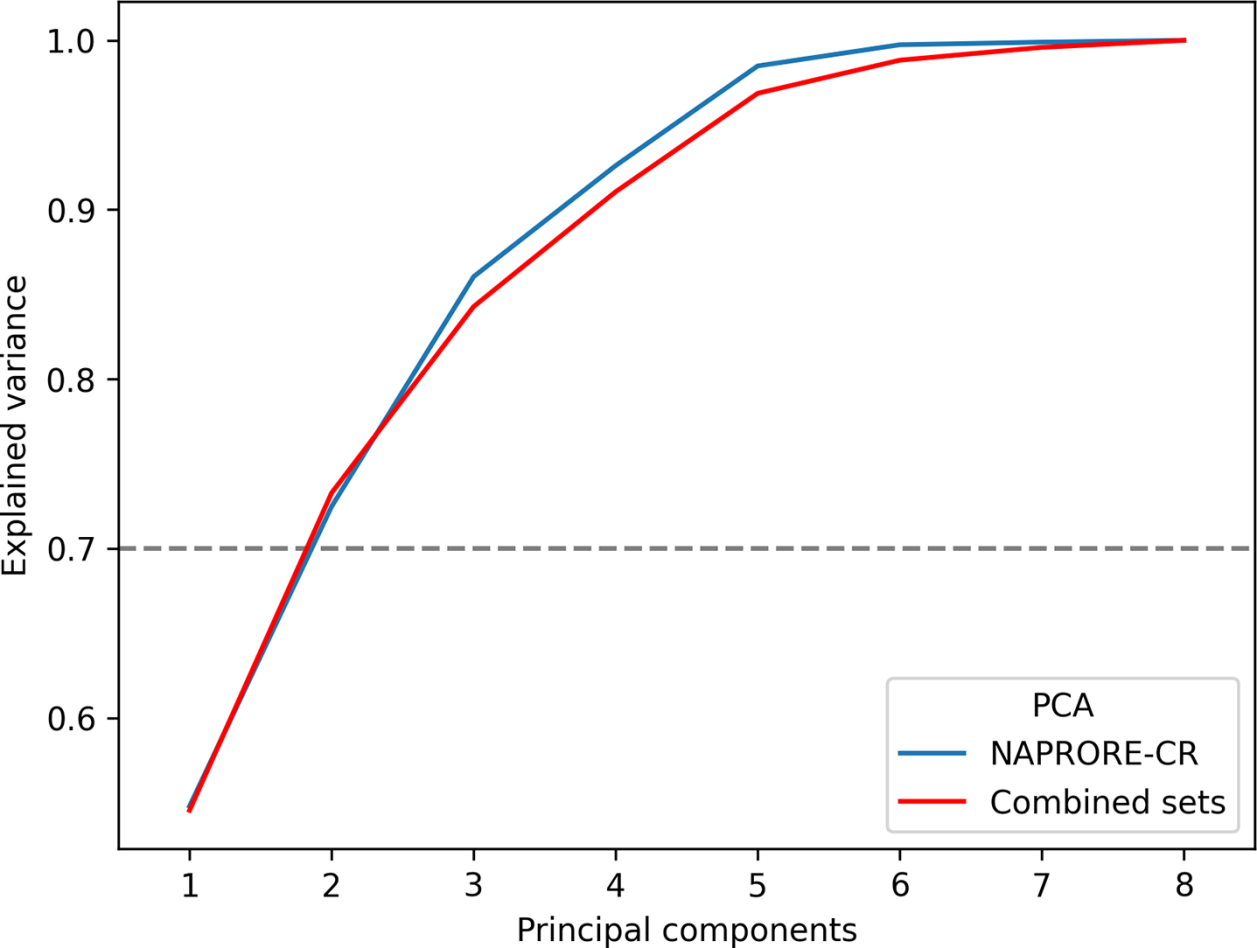
Cumulative explained variance in terms of the number of principal components generated for NAPRORE-CR and the combined data sets. The combined sets include NAPRORE-CR, approved drugs (DrugBank), natural pesticides (EPA), and cosmetic ingredients (COSMOS), which were obtained as described in Section 2.3.

Figure 8 shows the plotted result of the NAPRORE-CR PCA, with the respective biosynthetic routes for each compound denoted by the color of its marker. No clear separation between routes is observed, indicating that the properties of compound groups are not dissimilar. However, certain routes show visible tendencies; for instance, shikimate-phenylpropanoids extend horizontally, which suggests properties such as hydrogen bond donors/acceptors and TPSA describe their variance more efficiently. In contrast, fatty acids extend vertically, in the general direction of the AlogP and rotatable bonds property vectors, two important descriptors for this class of compounds (lipophilicity, unsaturations). Compounds located farther away from the central cluster correspond mainly to highly lipophilic terpenoids with multiple rotatable bonds or, conversely, to polyphenolic molecules with very high TPSA and hydrogen-bonding capacity. These structural extremes explain their displacement in the PCA space relative to the bulk of the data set. Other routes, such as polyketides, alkaloids, and amino acids and peptides, exhibit higher dispersion; however, as these groups have a low compound population, their tendencies are harder to determine.

**Figure 8:**
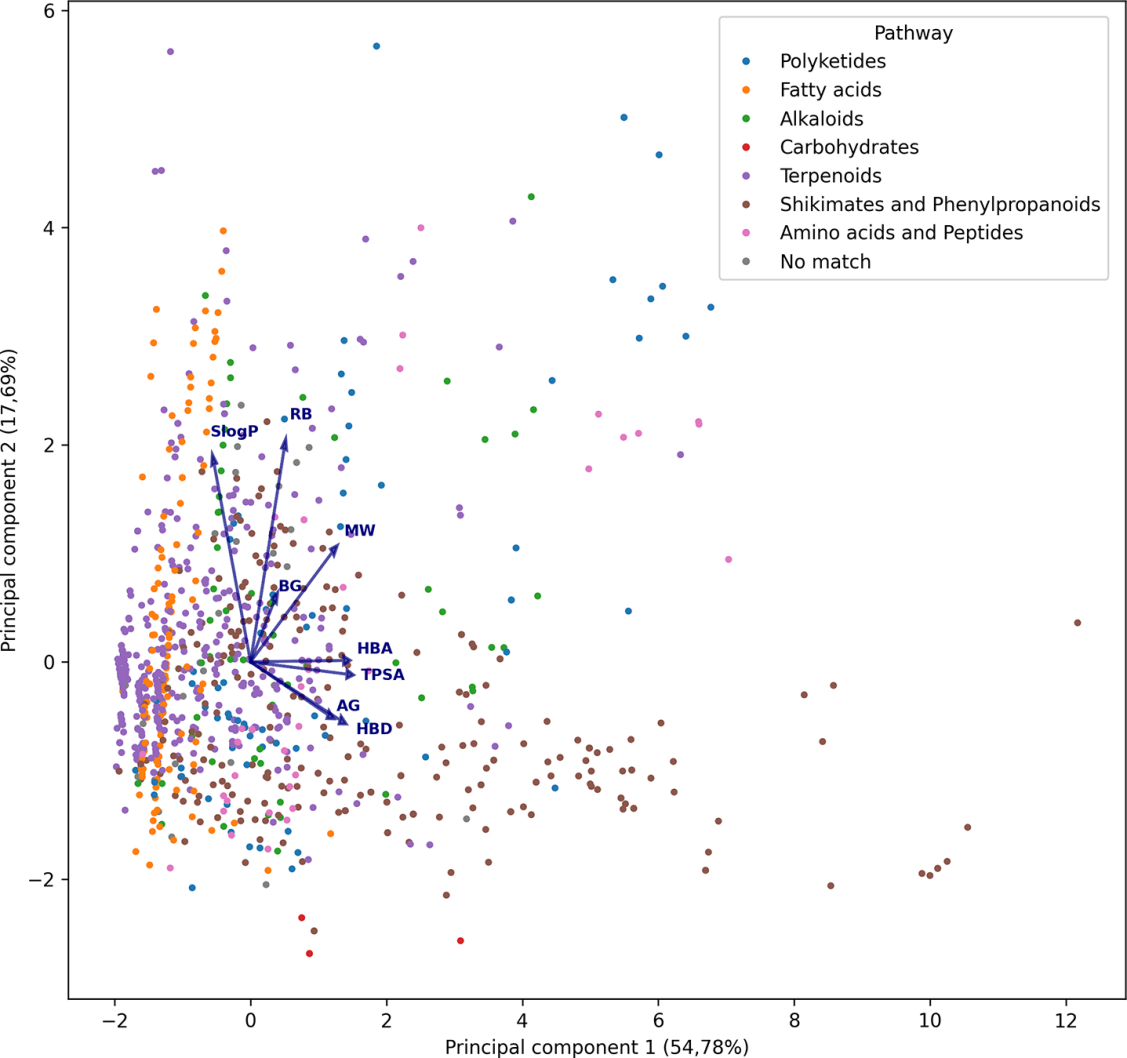
Chemical space visualization of the physicochemical and structural properties of NAPRORE-CR, generated by PCA (*n* = 1161 compounds). Vectors are scaled to represent the individual contributions of each property to the principal components.

The plotted PCA obtained from the combined datasets is shown in Figure 9, with individual subplots for each data set for readability purposes. The combined plot shows that the compounds of all four data sets occupy the same general property space with no clear separation between them. This suggests that the NAPRORE-CR compounds possess a physicochemical and structural property profile compatible for exploration in the drug, pesticide, and/or cosmetic ingredient spaces. Once again, the presence of NAPRORE-CR compounds (shikimate-phenylpropanoids) in a wide PC1 range is highlighted, which is a characteristic shared only with DrugBank. This is especially relevant when the size of these data sets is factored in: although COSMOS has a similar compound population to NAPRORE-CR, it does not show this same behavior in the PCA, which denotes a particularly high potential for exploring this compound pathway in the Costa Rica NPs space in the search for novel drugs, as they exhibit a similar property profile to compounds related to this field.

**Figure 9:**
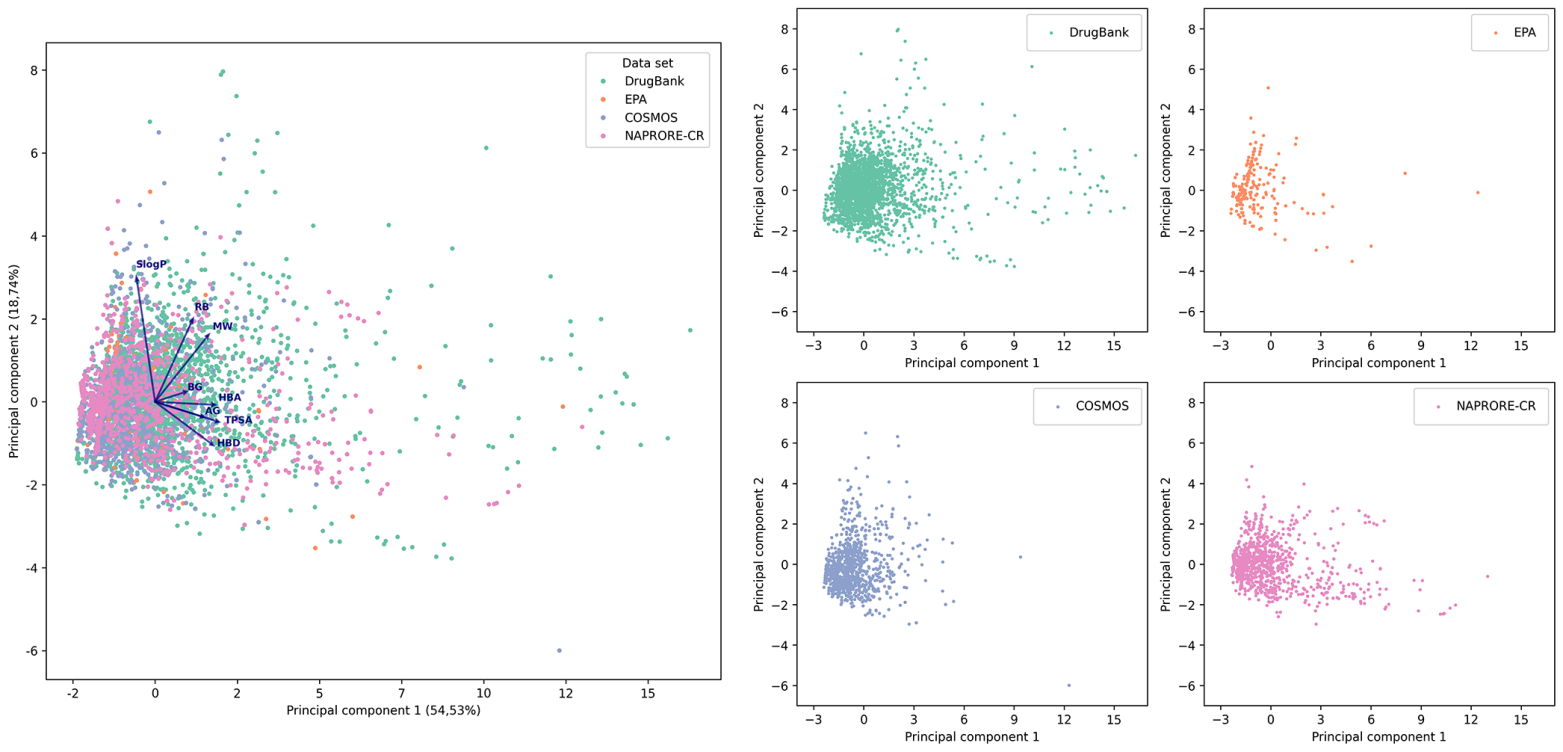
Chemical space visualization of the physicochemical and structural properties of DrugBank, EPA, COSMOS, and NAPRORE-CR, generated by PCA (*n* = 4963 compounds). Vectors are scaled to represent the individual contributions of each property to the principal components. The main plot encompasses all analyzed databases; additionally, a 2 × 2 grid displays individual plots for each data set.

#### 3.6.2 Structural Similarity Space (TMAP)

Figure 10 contains the plotted result of the generated TMAP for NAPRORE-CR, with the corresponding biosynthetic pathways of each compound represented by the color of its marker. Contrary to the PCA results, a clear separation by pathway can be observed, associated with the capability of molecular fingerprints to represent the structural characteristics of each compound family, which originates from their biotransformation processes and the metabolites that make up their structures.

**Figure 10:**
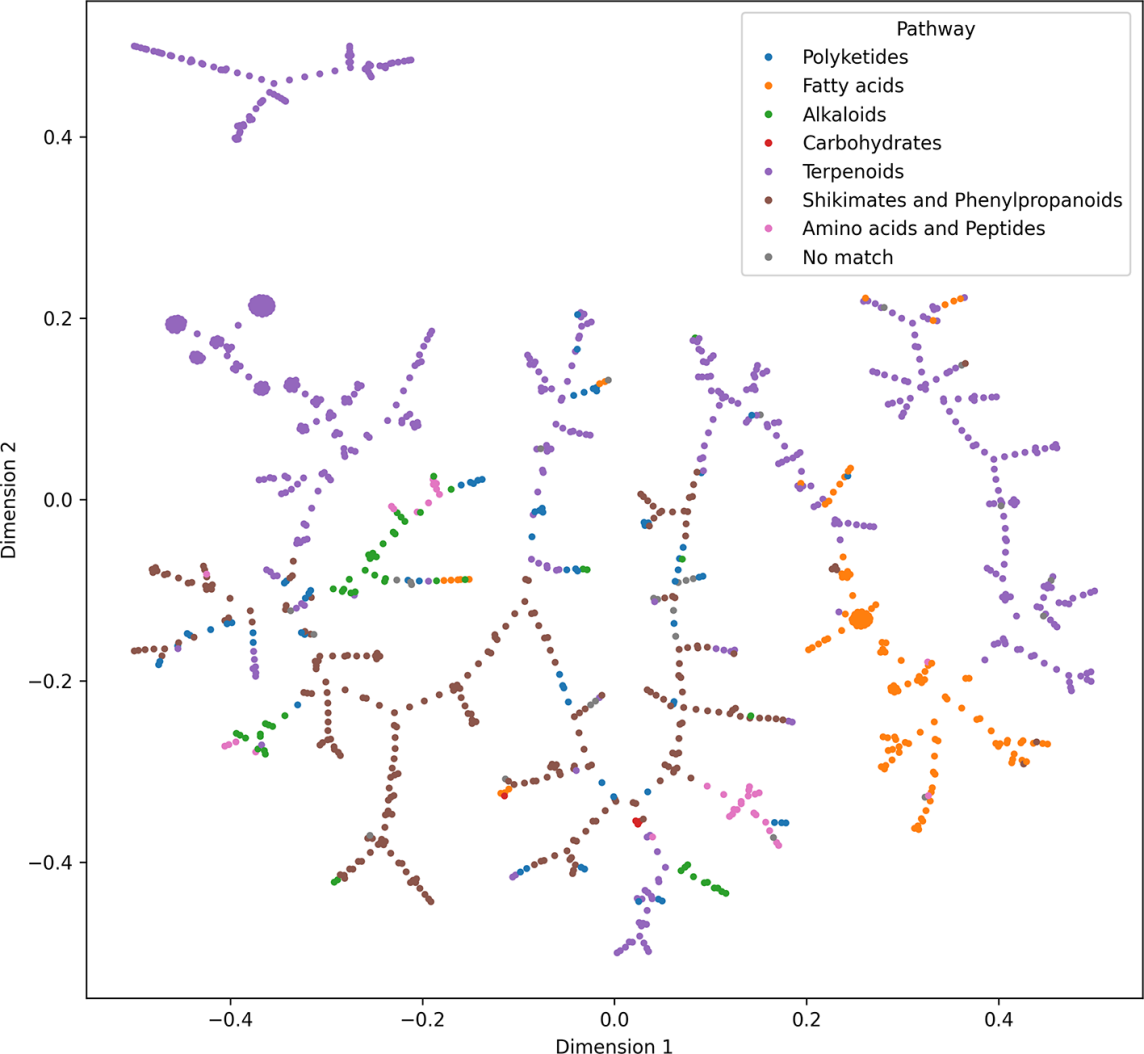
Chemical space visualization of the structural similarity based on MACCS Keys molecular fingerprints of NAPRORE-CR, generated by TMAP (*n* = 1161 compounds).

The pathways with the higher number of compounds in the database show more clearly defined branches that are independent of other groups, especially in the case of shikimate-phenylpropanoids and fatty acids, which have most of their compounds grouped in a single cluster. Since terpenoids have the highest share of representation by a large margin and exhibit great scaffold diversity, as mentioned previously, they are distributed across multiple branches, divided into other clusters, including a disconnected branch. This isolated cluster is composed mostly of terpenoid classes with low representation in the data set, such as aromadendrene, tujane, trichothecene, and cubebene sesquiterpenoids. Due to the low number of similar structures, the TMAP algorithm groups them in a cluster separate from the tree, which exemplifies its capability of isolating compounds with low structural similarity instead of forcing relationships with them. Polyketides are found dispersed throughout the entire tree, typically near shikimate-phenylpropanoid branches, with which they share scaffolds featuring multiple aromatic rings. Alkaloids share space with amino acids and peptides, due to the presence of nitrogen atoms in their structures; a small fraction of glycoalkaloids grouped with terpenic glycosides was also identified. Unclassified compounds were primarily found in phenylpropanoid branches, which coincides with the aforementioned observation about the nature of these structures in section 3.1. Overall, the TMAP visualization associated the less represented compound groups with other biosynthetic routes by their shared functional groups and similar scaffolds, which in turn might reveal a weakness of the MACCS keys system for this implementation, as it is based on predefined substructure dictionaries.

The TMAP for the combined sets is presented in Figure 11 and helps evaluate the structural similarity of the NAPRORE-CR compounds with the reference data sets. Once again, branches with a clear majority of compounds of a single set can be identified, especially for DrugBank and NAPRORE-CR, as these are the two biggest databases analyzed. NAPRORE-CR compounds are primarily located in the top right section of the plot, where they share space with most natural pesticides and a fraction of approved drugs; they also coincide with an EPA branch in the lower right quadrant (∼0.2 in dimension 1). The apparent dispersion of the COSMOS and DrugBank sets may be attributed to a higher structural diversity resulting from the presence of modified or synthetic molecules, compared to the entirely natural sets from NAPRORE-CR and EPA, which are comparatively more clustered in a single region of the space. Regardless, it is apparent that the NPs from Costa Rica share an important area of this chemical space with compounds used as natural pesticides or drugs, which suggests they possess a significant structural similarity with these data sets.

**Figure 11:**
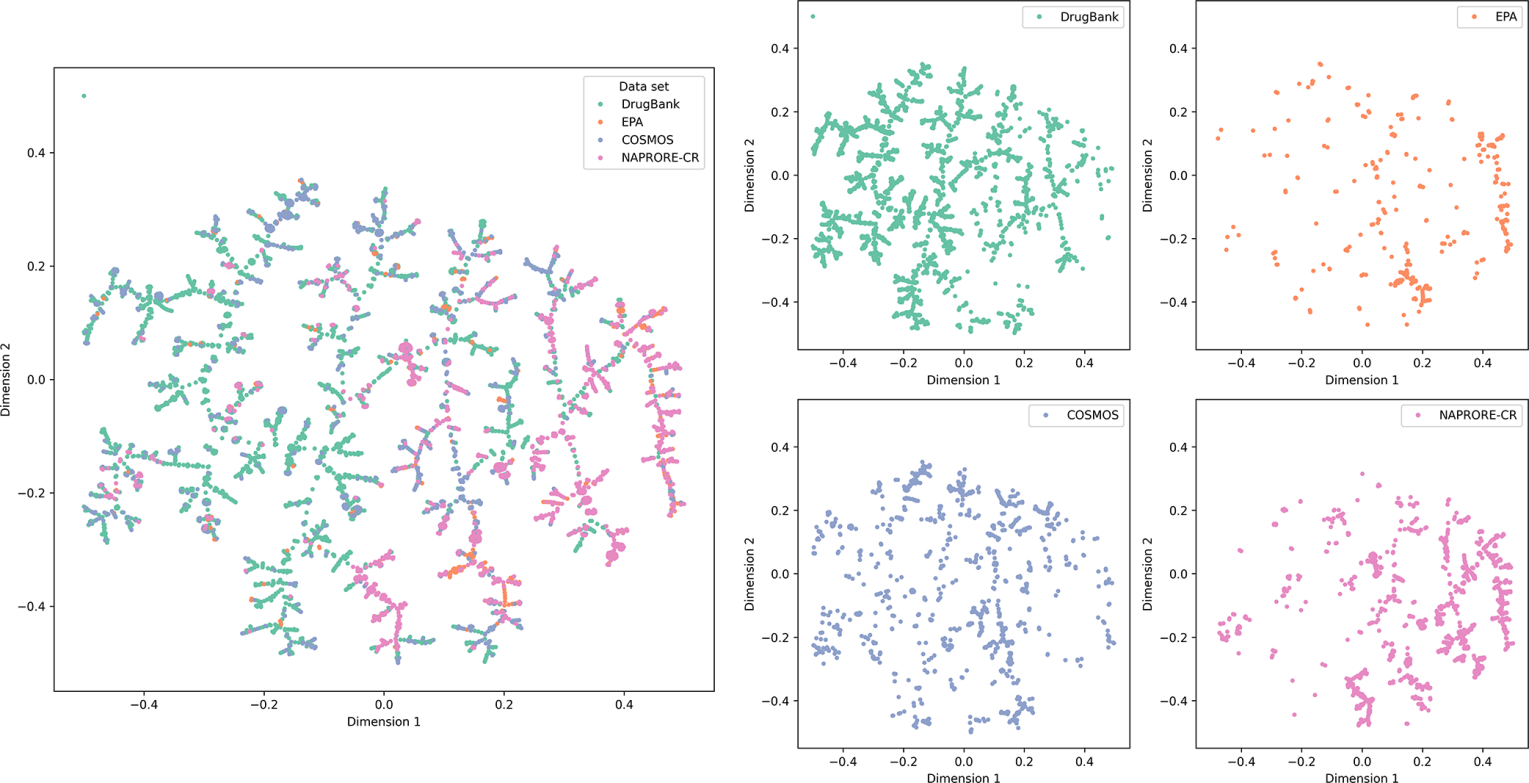
Chemical space visualization of the structural similarity based on MACCS Keys molecular fingerprints of DrugBank, EPA, COSMOS, and NAPRORE-CR, generated by TMAP (*n* = 4963 compounds). The main plot encompasses all analyzed databases; additionally, a 2 × 2 grid displays individual plots for each data set.

#### 3.6.3 Constellation Plot

Figure 12 shows the Constellation Plot generated for the NAPRORE-CR data set. Data points represent molecular scaffolds with at least two related compounds, while marker size indicates the number of related compounds. Data points are color-coded in a continuous scale from blue (low target promiscuity) to red (high target promiscuity); gray points indicate related compounds had no activity data. Selected chemical structures are highlighted to better visualize the relationships between the analog series. Out of 1100 identified cores, 873 were removed because they had only one related compound, leaving 227 cores related to 445 out of the 1161 compounds (38.33%). Since more than half of the compounds in the database lacked associated activity information in the ChEMBL database, a significant portion of this chemical space lacks promiscuity data. Three examples of this are the series on the bottom-left side (T), related to glycosylated tryptophan alkaloids, the series on the bottom-right side (K), composed of kaurane diterpenoids, and the series on the top-center side (G), formed by germacrane sesquiterpenoids, the single most represented compound class in the database. The absence of activity data could represent an opportunity to discover applications for unexplored compound families.

**Figure 12:**
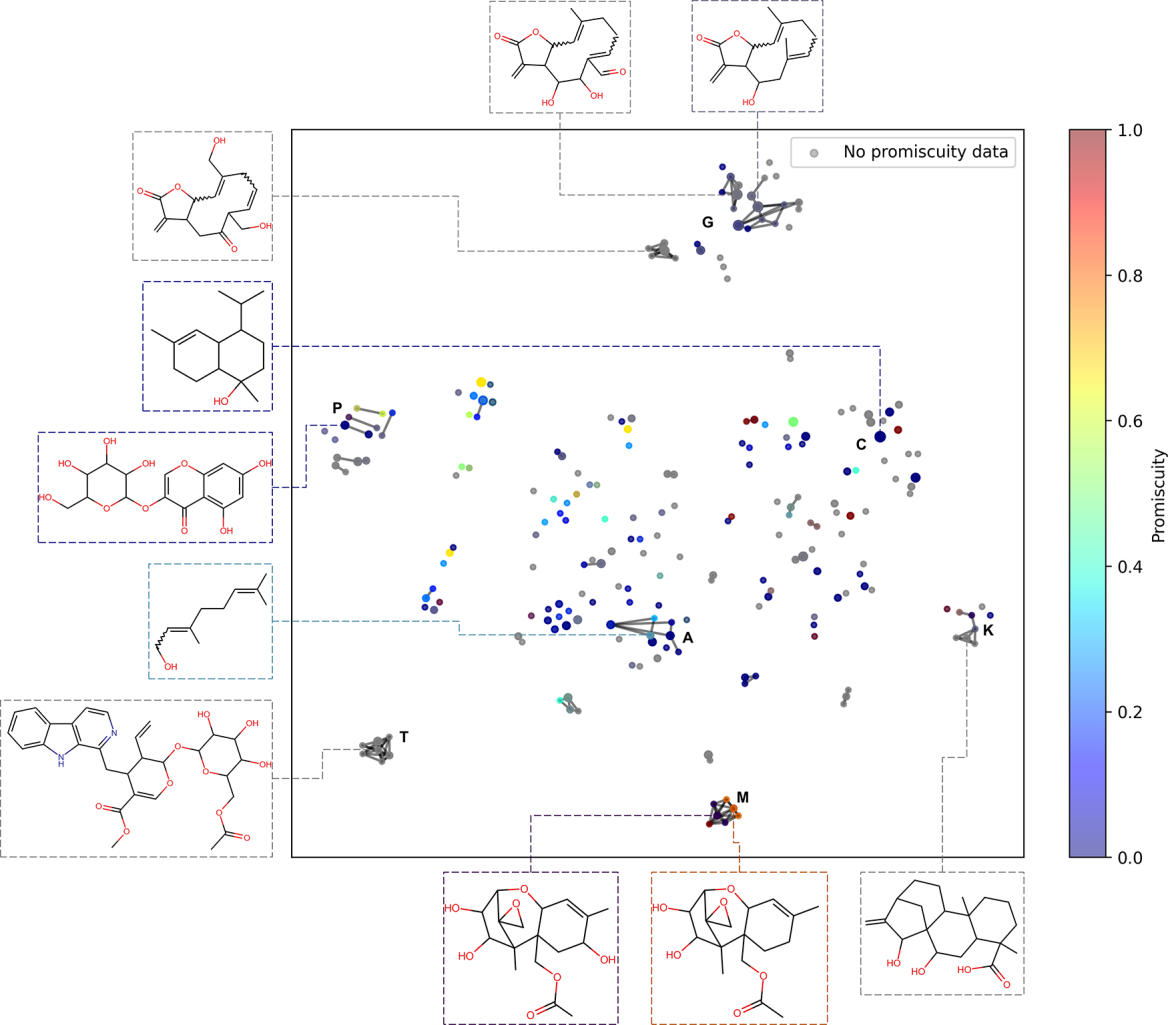
Constellation plot for the NAPRORE-CR data set. Marker size corresponds to the number of related compounds. Color scale indicates the promiscuity ratio calculated from activity assay information retrieved from ChEMBL; gray points indicate no activity data.

A noteworthy series is the one found at the bottom-center of the plot (M), represented by a trichothecene sesquiterpenoid scaffold and corresponding to a series of mycotoxins, which exhibits significant promiscuity differences caused by the addition or removal of hydroxyl groups and/or acetyl/hydroxyl group replacements. This apparent promiscuity cliff exemplifies the utility of Constellation Plots for exploring the relationships between structural analog series and activity,^85^ and it represents a possible research line worth considering in future studies. Another notable series is located at the midcenter of the plot (A). It is one of the most numerous, as it is related to acyclic monoterpenoids, one of the most abundant classes of compounds in the database. Similarly, marker C corresponds to cadinane sesquiterpenoids. The clustered pairs on the midleft area (P) correspond to glycosylated aromatic polycycles, such as polyketides and flavonoids, with low to middling promiscuities. As these compound groups have shown great importance in the discovery of antitumoral, antimicrobial, antiparasitic, and other classes of medicinal agents,^107,108^ expanding on these pathways could prove promising for the discovery of selective drug-like entities.

### 3.7 Global Diversity Analysis

The cyclic system recovery (CSR) curves, generated to quantify scaffold diversity in the four studied data sets, are shown in Figure 13. These plots suggest that the sets with the highest scaffold diversity are DrugBank and NAPRORE-CR, as these two have the lowest areas under their respective curves compared to EPA and COSMOS. Using this measure, a data set with a unique scaffold for every molecule (maximum diversity) would result in a straight line in the CSR plot and an AUC of 0.5, while less diverse sets would show higher values.^90^

**Figure 13:**
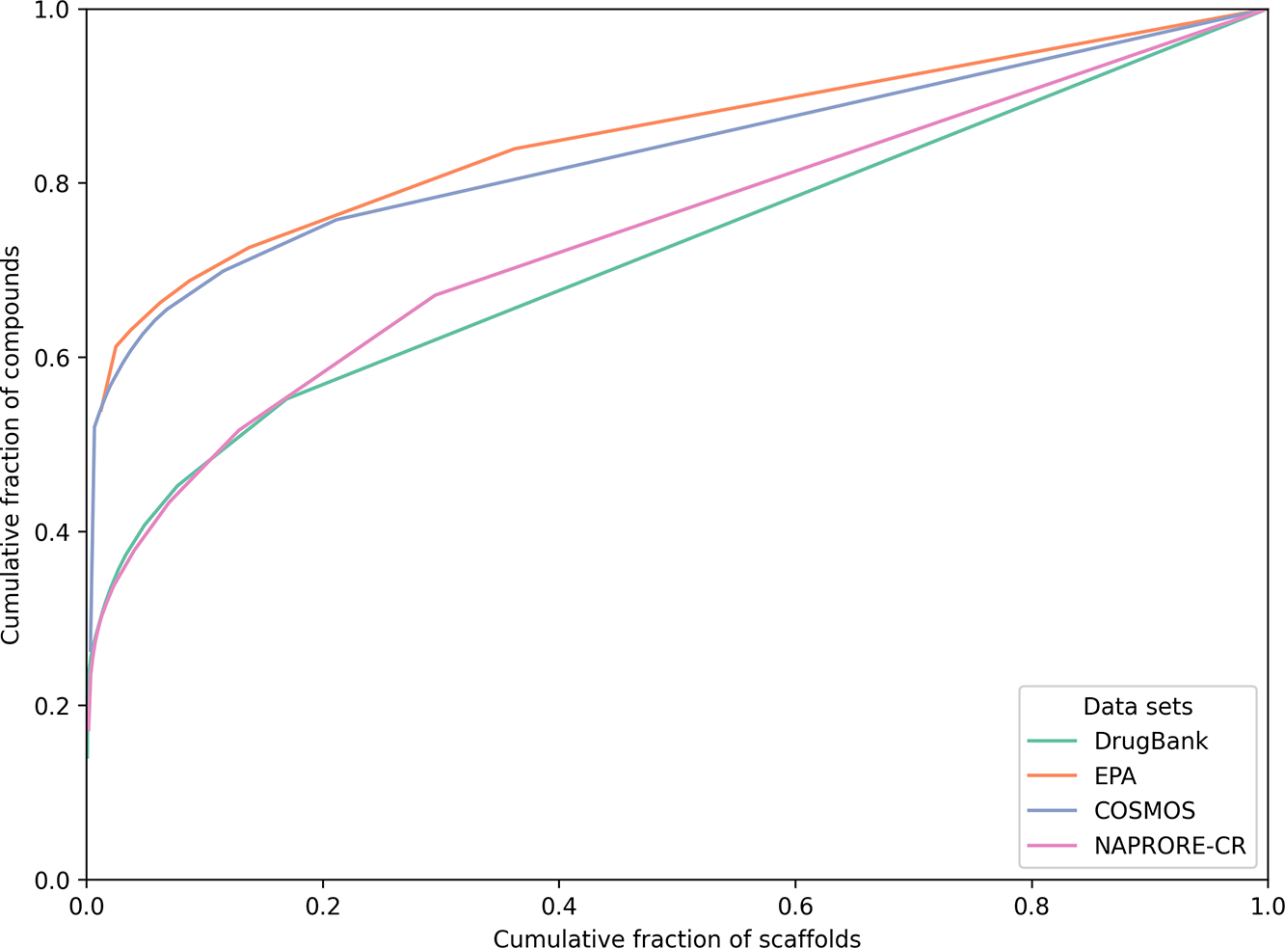
Cyclic recovery system curves for the calculation of a scaffold diversity metric for the analyzed compound databases.

The CDP obtained from the intraset Tanimoto coefficient and AUC calculations for every set is shown in Figure 14. As the Tanimoto coefficient measures structural similarity, lower values are expected for more diverse compound sets. The CDP shows that NAPRORE-CR and DrugBank possess a higher scaffold diversity and a lower molecular fingerprint diversity (NAPRORE-CR: mean Tanimoto coefficient = 0.32; AUC = 0.74. DrugBank: mean Tanimoto coefficient = 0.28; AUC = 0.72) compared with COSMOS and EPA (COSMOS: mean Tanimoto coefficient = 0.18; AUC = 0,83. EPA: mean Tanimoto coefficient = 0.24; AUC = 0.85). This suggests that the Costa Rican natural products space exhibits structural diversity comparable to that of the approved drugs space, despite being considerably smaller in terms of the number of compounds. The primary difference between these two sets lies in the higher molecular fingerprint diversity in DrugBank, which can be attributed to the aforementioned structural modifications commonly applied to drug candidates to enhance their properties, bioavailability, and biological activity.

**Figure 14:**
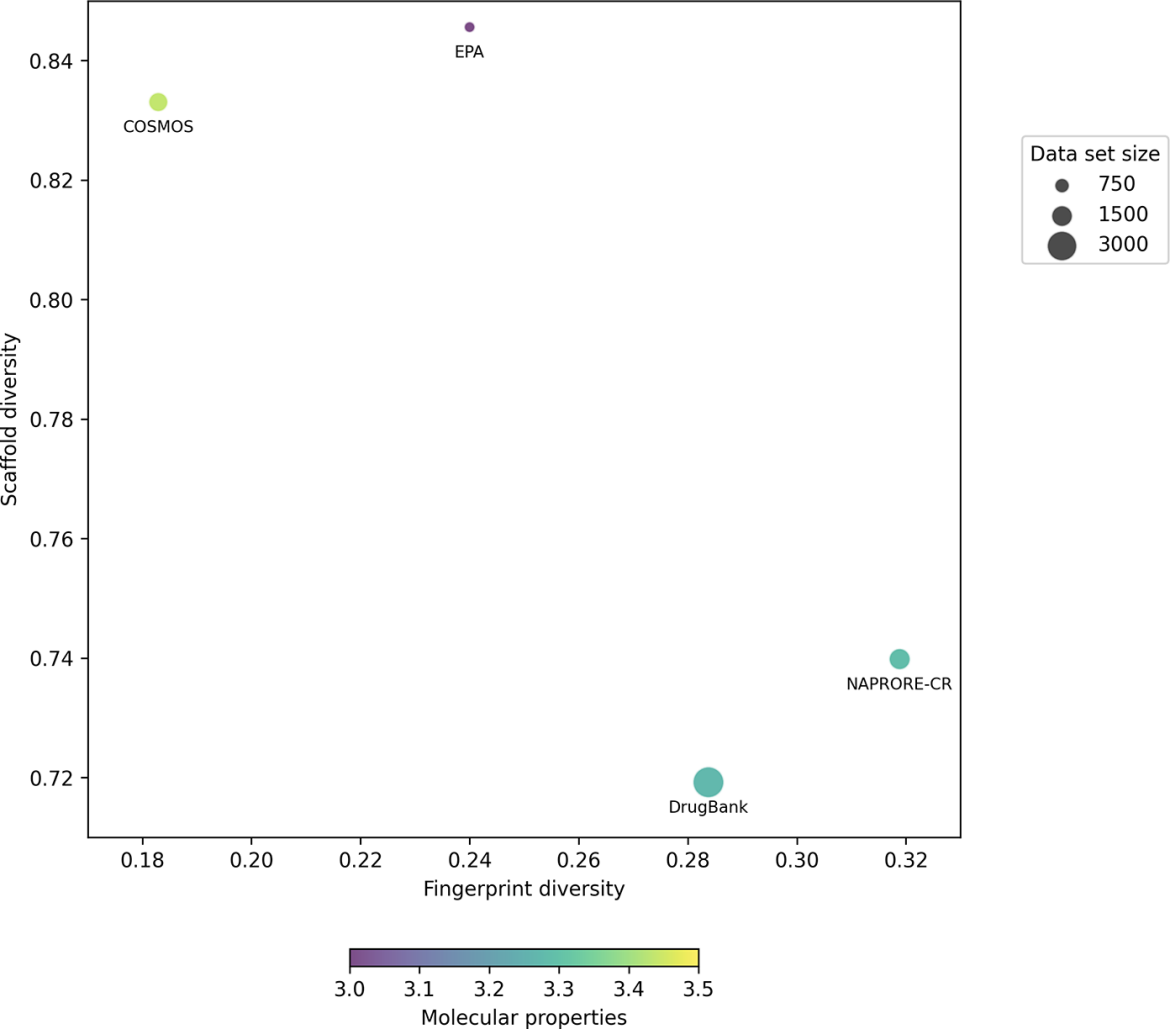
Consensus diversity plot of DrugBank, EPA, COSMOS, and NAPRORE-CR, based on MACCS Keys molecular fingerprints and Murcko molecular scaffolds. *X*-axis values represent molecular fingerprint diversity (mean Tanimoto coefficient, with lower values indicating higher diversity), and *Y*-axis values correspond to molecular scaffold diversity (AUC from the cyclic recovery system curves, with lower values indicating higher diversity). Marker size represents the number of compounds in each data set, while the continuous color scale relates to the physicochemical and structural properties calculated in Section 3.3.1 (mean Euclidean intraset distance).

The COSMOS data set, although of similar size to NAPRORE-CR, exhibits higher molecular fingerprint diversity, attributed to the variety of types and sources of compounds included in the database (botanical, animal-derived, synthetic, among others). For instance, COSMOS also includes halogenated species, siloxanes, and other substances with heteroatoms not present in NAPRORE-CR. The lower scaffold diversity of COSMOS can be associated with a higher proportion of molecules that have an aliphatic chain or benzene as their scaffold. These two structures represent more than half of the molecules in COSMOS (51.9%), versus 24.0% for DrugBank and 23.6% for NAPRORE-CR. The EPA data set shares these characteristics with COSMOS: higher source diversity compared to NAPRORE-CR (vegetal, animal, microorganisms) and lower scaffold diversity dominated by the same two scaffolds (61.6% of its compounds, with 54.4% corresponding to aliphatic chains), although this case is harder to assess due to the low sample size.

## 4 Conclusions

The first version of the Natural Products repository of Costa Rica was introduced, featuring 1161 compounds obtained from 105 manuscripts, including scientific papers and theses from the country’s leading public universities and research centers dedicated to the study of NPs. Thus, NAPRORE-CR aligns with open science initiatives and adheres to the FAIR principles for data sharing, making the rich molecular diversity of Costa Rica’s biodiversity accessible to the global scientific community.

According to the NPClassifier service, the most frequent compound families in the database are terpenoids, shikimates and phenylpropanoids, and fatty acids; their corresponding superclasses and classes demonstrate the predominance of plants as the primary source of compounds included in this resource. The physicochemical and structural profiles of the data set showed similarities with the COSMOS cosmetic ingredients database in terms of molecular size, flexibility and polarity, and with EPA natural pesticides in the MW and AlogP properties. Overall, most of the NAPRORE-CR data set complies with empirical rules for drug oral bioavailability (Lipinski’s Ro5). Calculated complexity descriptors indicate that although the structural complexity associated with NPs might prove challenging, the space contains a significant amount of synthetically feasible, potentially bioactive compounds for medicinal chemistry applications. Cross-referencing NAPRORE-CR with the PubChem and ChEMBL databases revealed that 51.59% of its compounds are commercially available, and that 49.70% have associated bioactivity assay data; however, only 24.26% of these compounds showed positive activity results. Predicted target affinity suggests that the nuclear hormone receptor family is a potential area of study of the Costa Rican NPs space, with reported experimental evidence of compounds present in NAPRORE-CR that exhibit activity as treatments for prostate cancer.

The visual representation of the chemical space with PCA showed that NAPRORE-CR shares a similar property space with all the analyzed reference data sets (DrugBank, COSMOS, EPA), with a notable tendency for the shikimate-phenylpropanoids pathway to extend into a region of space only shared with approved drugs. The TMAP visualization revealed structural similarities with compounds used as natural pesticides or drugs, suggesting the possibility of deriving analog structures with applications in these fields. The Constellation Plot highlighted analog series with potential for further study, including an apparent promiscuity cliff in a series of mycotoxins, as well as several polyketides and flavonoids with low target promiscuity. The consensus diversity plot revealed that the NAPRORE-CR database is comparable to the DrugBank database in terms of scaffold, fingerprint, and property diversity, despite being significantly smaller in terms of the number of compounds. We expect NAPRORE-CR to aid current work and inspire future research lines for the rational utilization of the country’s natural wealth.

## Data Availability

All the dataset files used to generate the plots and chemical space visualizations for NAPRORE-CR and the compiled EPA pesticides list are freely available at the corresponding Zenodo repository: 10.5281/zenodo.7858061. In this first version, the data set is distributed as a CSV file, which can be easily accessed and processed with common tools (e.g., Python, R). We are currently exploring the development of a dedicated webpage to provide a more user-friendly and visual access to the database in future updates. The COSMOS data set can be freely obtained through its website after registration: https://cosmosdb.eu/cosmosdb.v2. The DrugBank data set can be accessed for academic purposes by applying for a license through the official website: https://go.drugbank.com. The Python scripts built to generate the plots and visualizations can be freely obtained from the CBio^3^ Group’s GitHub repository: https://github.com/cbio3lab/.

## Abbreviations:

CADD: Computer-aided drug design
EPA: United States Environmental Protection Agency
FAIR: Findable, Accessible, Interoperable, and Reusable
FDA: United States Food and Drug Administration
NAPRORE-CR: Natural Products Repository of Costa Rica
NPs: Natural products
PCA: Principal component analysis
t-SNE: t-distributed Stochastic Neighbor Embedding
QSAR: Quantitative structure–activity relationship models
TMAPs: Tree MAPs
VS: Virtual screening
